# Clinician-informed XAI evaluation checklist with metrics (CLIX-M) for AI-powered clinical decision support systems

**DOI:** 10.1038/s41746-025-01764-2

**Published:** 2025-06-14

**Authors:** Aida Brankovic, David Cook, Jessica Rahman, Alana Delaforce, Jane Li, Farah Magrabi, Federico Cabitza, Enrico Coiera, DanaKai Bradford

**Affiliations:** 1 CSIRO’s Australian eHealth Research Centre, Herston, QLD Australia; 2 The University of Queensland, Brisbane, QLD Australia; 3 Princess Alexandra Hospital, Brisbane, QLD Australia; 4 Macquarie University, Sydney, NSW Australia; 5 Australian Institute of Health Innovation, Sydney, NSW Australia; 6 University of Milano-Bicocca, Milan, Italy; 7 IRCCS Ospedale Galeazzi - Sant’Ambrogio, Milan, Italy

**Keywords:** Translational research, Publishing

## Abstract

The rapid growth of clinical explainable AI (XAI) models raised concerns over unclear purposes and false hope regarding explanations. Currently, no standardised metrics exist for XAI evaluation. We developed a clinician-informed, 14-item checklist including clinical, machine and decision attributes. This is the first step toward XAI standardisation and transparent reporting XAI methods to enhance trust, reduce risks, foster AI adoption, and improve decisions to determine the true clinical potential of applied XAI.

## Introduction

Artificial intelligence (AI)-supported clinical decision support systems (CDSS) are seen as a promising solution for improving efficiency, accuracy, and cost-effectiveness in medical decision-making processes^1,2^. Despite the potential benefits, the “black box” problem, where users are not privy to the AI decision-making process, fuels scepticism, especially in high-stake environments where trust is non-negotiable. Safety and transparency are among the two most critical issues influencing every aspect of AI-based medical decision-making, playing a key role in establishing trust and acceptance of the technology^3,4^.

Explainable AI (XAI) has been seen as an important factor in promoting transparency and enhancing user confidence, contributing to more trustworthy AI systems. Post hoc XAI methods do not reliably reveal the causal pathways or inner workings of intrinsically black-box algorithms. Some studies argue that non-explainable AI should be prohibited in healthcare^5^. Recent studies, however, report that explanations can sometimes harm decision making^6^ and may mislead clinicians^7^. Additionally, explanations obtained by different state-of-the-art XAI methods may provide divergent or inconsistent data and disagree^8,9^. The presence of erroneous or inconsistent explanations could lead to fatal errors when using CDS tools, jeopardising the core ethical principle in medicine to ‘first, do no harm’^8^. These shortcomings compromise key aspects of the criteria identified as crucial for effective translation to clinical practice and building trust in ML models^10^, which includes domain appropriate representation, actionability and consistency.

Several AI reporting checklists have come into widespread use, including TRIPOD^11^, TRIPOD + AI^12^, CLAIM^13^, to understand, evaluate, replicate, and apply the models appropriately. However, each fall short in fully addressing the nuances of explainability, for instance how explanations should be evaluated, contextualised, and used in clinical practice despite exponential growth in the number of studies of applying XAIs^14^. While a significant body of research leverages XAI to meet regulatory requirements and foster trust in AI-supported CDS, there are still no available standardised metrics and XAI evaluation guidelines^15^ informed by domain experts or users. The development of such an evaluation framework is recognized as a priority in the *XAI Manifesto 2.0*^15^, a comprehensive roadmap for advancing the field of XAI. Several recent studies^10,16–21^ provided conceptual value and contextualised XAI for clinical use, though they do not offer a structured or evaluative tool that can be directly used to guide the development or review of clinical XAI applications. Nauta et al.^14^ provided an extensive overview of quantitative XAI evaluation methods used in the XAI literature and identifies 12 properties to evaluate explanations without user studies. Clinical XAI guidelines intended for medical image analysis comprising five criteria to support the design and evaluation recommendations of clinically viable XAI are proposed by Jin et al.^22^. It is modality specific, focused on clinical attributes only and lacks qualitative and quantitative assessments that reflect real-world clinical effectiveness for the considered case.

To bridge the gap and aid authors and reviewers of AI manuscripts in health informatics, we have developed a Clinician-Informed XAI Evaluation Checklist with metrics (CLIX-M) (Table 1, Supplementary Table 1). The checklist is intended for the development and evaluation phases of XAI component in AI-powered CDSS development. The checklist was informed by a qualitative study undertaken by the Australian e-Health Research Centre, looking at mitigating ethical risks in development of AI enabled clinical tools with an XAI component. Full methodology is provided elsewhere^23^. We also drew on recent relevant studies^9,10,14,17,19^ sourced from PubMed using search terms ‘XAI’, ‘Explainable AI’ and related synonyms. We merged these into a checklist that aligns with the EQUATOR Network guidelines^24^. The checklist covers 4 categories (*Purpose*, *Clinical Attributes*, *Decision attributes*, *Model Attributes*), comprising a total of 14 items, with accompanying recommendations and justifications. Each category’s items are discussed with suggested metrics, notes, and examples to illustrate key aspects. The recommendations are not exclusive and may vary depending on the CDSS being developed. Section where each item should be reported i.e. methods (M), results (R) and/or discussion (D), and whether it applies to the development (D) and/or clinical evaluation (E) phase is included (Tables 1 and 2, Supplementary Tables 1 and 2)The items described here should be viewed as a clinician-informed guide for authors and relevant stakeholders (Table 3) in evaluating and reporting deployed XAI. To illustrate how each evaluation consideration can be applied in practice, hypothetical examples of AI applications being evaluated are provided in the Supplementary Tables 3 and 4. The checklist and its recommendations form a provisional guide that can be used to establish wider consensus and enhance the checklist’s applicability across diverse clinical domains and use cases and inform the development of broadly standardised XAI checklist through a Delphi panel process.

**Table 1:** CLIX-M checklist for the evaluation and reporting of studies including eXplainable AI (XAI)

Item	Checklist item	Item details	Phase^a^	Section^b^
1	Purpose	Provide a summary of objectives, i.e. for which purpose explanations are developed and intended.	D;E	M
Clinical attributes				
2	Domain relevance	Assess (see Table 2), report and discuss explanation actionability performed by the end users or the relevant domain literature.	D;E	M;R;D
3	Reasonableness	Assess (see Table 2), report and discuss how the explanations agree with human rationales and how reasonable they are.	D;E	M;R;D
4	Actionability	Assess (see Table 2), report and discuss explanation informativeness and potential to impact the workflow.	D;E	M;R;D
Decision attributes				
5	Correctness	Assess, report and discuss the correctness of explanations benchmarked against the ground truth as it is done for quantifying predictive accuracy of a model	D;E	M;R;D
6	Confidence	Quantify, report and discuss confidence of deployed XAIs, i.e. explanations’ confidence scores.	D;E	M;R;D
7	Consistency	Quantify, report and discuss XAIs 1) sensitivity on underlying design variations, 2) sensitivity on the deployed model by quantifying feature agreement at cohort level (if applicable) and 3) agreement on contribution direction by quantifying sign agreement at the patient level.	D;E	M;R;D
8	XAI robustness	If ensemble XAI is used to improve explanation robustness provide details which methods are used and how final explanations are obtained.	D;E	M;R;D
9	Causal validity	Discuss capability of deployed XAI to capture causal relationships. If it capable to capture casual relationships provide relevant details and how it is validated.	D;E	D
Model attributes				
10	Narrative reasoning	Explore and report informative relationships between explanations and the patients’ trajectories (if appliable).	D;E	R;D
11	Bias and Fairness	Report how explanations are used to explore model fairness and potential biases. Explain the findings, and whether it is used for further model improvement.	D;E	R;D
12	Model troubleshooting	Explore data distribution of the main contributors for correct and incorrect predictions/decisions (TP, TN, FP, FN)^c^. Report overlaps between correct and incorrect outputs and whether and how this information has been used for model improvement.	D;E	R;D
13	Interpretation	Provide an overall interpretation of the main findings in the context of XAI and model audits, and related prior research.	D;E	D
14	XAI limitations	Discuss XAI limitations.	D;E	D

When using XAI in practice: a. Use clinical judgement to evaluate explanation coherence. b. Be aware of XAI limitations. c. Be aware of data quality used by XAI system. d. Treat XAI output only as an adjunct.

^a^D = items relevant only to XAI development; E = items relevant to the evaluation of a XAI in clinical settings.

^b^M = items to be reported in the Methods section; R = items to be reported in the Result section; D = items to be reported in the Discussion section (if meaningful).

^c^True positive (TP), true negative (TN), false positive (FP), false negative (FN).

**Table 2:** Scales for clinical attributes: domain relevance, reasonableness, actionability

Domain relevance	Coherent	Actionability
Very irrelevant	Very incoherent	Not actionable
Irrelevant	Incoherent	Slightly actionable^a^
Relevant	Coherent	Actionable
Very relevant	Very coherent	Highly actionable

^a^Vague or limited usefulness to action; might support decision-making but not reliably. For example, not all highlighted image areas are clinically meaningful or impact the workflow. However, some useful information can still be extracted and acted upon.

**Table 3:** Stakeholder roles and their engagement with the CLIX-M

Stakeholder	Role and engagement with the CLIX-M	
Clinicians/Physicians	Evaluation of clinical attributes	Evaluate whether explanations align with clinical reasoning and support safe, informed decisions.
Data Scientists/ML practitioners	Evaluation in the context of decision and model attributes	Implement and validate XAI methods; ensure explanations are technically sound and accurate.
UX/UI Designers	Evaluation relevant to inform UX/UI design	Leverage CLIX-M to guide the design of explanation delivery mechanisms that are both intuitive and contextually appropriate.
Regulators/QA	Auditability, comprehensiveness of documentation, compliance	Use CLIX-M to review adherence to standards and evaluate explainability for accountability and safety.

## Clinician-informed XAI evaluation checklist with metrics (CLIX-M)

### Purpose

***Item 1****. Purpose of deploying:* Explain for which purpose explanations are developed and intended^25^, e.g. understand model rationale and to identify key contributors, investigate model biases, perform model trouble shooting. XAI encompasses a broad range of methods, including feature attribution, saliency maps (SM), counterfactual and natural language (NL) explanations^18^ and purpose of deploying XAI can help choice of method. FA and SM-based approaches map explanations onto familiar clinical concepts, offering fast, intuitive outputs critical in time-sensitive settings such as ICU and facilitate model auditing and debugging. Counterfactuals are valuable for supporting “what-if” reasoning, although their utility may be limited in urgent or high-stakes settings where cognitive load and timeliness are concerns^10^.

### Clinical attributes

Plausible explanations can either reinforce or challenge a clinician’s existing perspective and interpretation^23^. To evaluate the plausibility of explanations, developers or users should assess how well the explanation aligns with the clinician’s mental model-specifically in terms of domain relevance (i.e., pertinence to the clinical task), coherence, and actionability^10,22,23^-within the context of the specific case at hand (Table 2).

To support systematic evaluation, the checklist is supplemented with Likert-type^26,27^ rating scales. Each clinical attribute captures a distinct concept, thus an aggregated score across all clinical attributes is not meaningful except to give an overall impression of useability. Using rating scales during checklist application can help standardise judgments and facilitate comparisons across models or users. For instance, a clinician might rate actionability on a 4-point scale ranging from “Not actionable at all” to “Highly actionable and directly supports clinical decision-making.” Similarly, coherence can be rated based on how well the explanation aligns with plausible clinical reasoning for the case at hand. Analysing response items should be done categorically as suggested by Boone^27^. The threshold for considering a variable relevant/reasonable/actionable depends on the intended purpose or use case. Expert reasoning can vary, thus involving multiple domain experts is recommended. Aggregating responses and using ordinal descriptive statistics^27^ helps quantify consensus.

***Item 2****. Domain relevance:* Explanations should be domain-appropriate for the application task^10,16^, avoiding redundancy or confusion. Key contributing factors should be evaluated during development and implementation, scored *Very irrelevant* to *Very relevant* based on alignment with domain knowledge and consensus (Table 2). The assessment should be guided by Grice’s maxims of quality, quantity, relevance, and clarity^28^. Only explanations relevant to the application at hand are clinically useful.

For tabular data relevance at the cohort level can be assessed by the developer though summing feature importance-frequently important local factors should rank highly. For images, relevance can be evaluated at the cohort level by calculating the hit rate, which measures alignment with known relevant regions^29^. However, counterfactual explanations are harder to validate, as their domain relevance depends on the plausibility and clinical validity of the proposed changes. Overall, explanations should align with expert knowledge and/or established literature.

***Item 3****. Coherence:* Evaluates how well the explanation aligns with relevant background knowledge, expert beliefs, and established clinical consensus, thereby addressing concepts such as reasonableness, plausibility, and agreement with human rationales^14^. Explanations which match clinicians’ reasoning boost trust^23^. Retrospective studies can be valuable in conducting patient-level analyses by checking local explanations for each patient to assess alignment with human reasoning. End users should evaluate this during development and testing, using 4-point Likert scoring (see Table 2).

***Item 4****. Actionability:* Reflects the explanation’s ability to support downstream clinical decision-making by enabling the user to take safe, informed, and contextually appropriate actions based on the information provided^10^. Explanations should be timely, informative and impactful - guiding clinical workflows or suggesting actions. In settings where rapid decision-making is critical such as ICU, counterfactual explanations may be impractical, as they often lack immediate usability and can contribute to cognitive overload^10^.

While modifiable, causative factors are most valuable, unmodifiable or associative factors still hold some value for action. Explanations that are actionable are clinically most useful and actionability should align with human reasoning. For example, high LoS, where the patient has been in the hospital for a long time, indicates a higher risk of deterioration in general terms, however, this variable is less clinically useful as there is no action available for clinicians.

Patient-level analysis, involving clinical partners, should be performed during development to evaluate explanation informativeness and workflow impact using scoring system (Table 2). In case of counterfactual explanations, actionable counterfactuals should propose changes that are clinically plausible and relevant to decision-making.

Note: Only highly relevant, actionable variables should be displayed on dashboards intended for end-users. Other variables should support modelling or prediction; or serve as optional context if beneficial but non-essential (e.g., LoS).

## Decision attributes

***Item 5****. Correctness:* It represents the fraction of correct explanations relative to the total number of evaluated samples. Local explanations can be compared to ground truth (where available), measuring correctness in the same way as for model accuracy^9^. For imaging data, mIoU and hit rate^29^ can be used. In contrast, evaluating decision attributes for counterfactual explanations is more challenging; focusing on clinical attributes may be more appropriate. Systematic agreement with clinical causes increases trust^23^.

***Item 6****. Confidence:* Confidence concerns whether the explanations has a measure of certainty or other probability information^14^. Explanations, like predictions, can be sensitive to input perturbations, data errors or model instability. Knowing the machine’s confidence associated with its explanation boosts clinicians’ trust^23^. To quantify explanation of uncertainty and/or confidence intervals we recommend the following approaches used for calculating model confidence intervals^30^:

*Bootstrapping:* Resample the input data (e.g., 70%) and compute global explanations for each resampled dataset. Aggregate the rankings and explanation values to calculate the mean and confidence interval for feature importance or hits.

*Repeated Model Training:* For non-deterministic models (e.g., neural networks, random forests), train multiple versions with different seeds and subsamples to assess sensitivity. Calculate global feature importance and confidence intervals across models.

Note: Feature rankings and explanation values are correlated, with higher-ranked features contributing more significantly to the model’s output. For comprehensive insights, both should be quantified. While bootstrapping offers a faster way to estimate variability—primarily reflecting data sampling effects—repeated model training provides a more robust view of model’s robustness. In tree-based models, uncertainty can be quantified by averaging feature rankings across individual trees and calculating both the mean and confidence intervals for each feature’s contribution.

***Item 7****. Consistency:* Consistency checks ensure that identical inputs lead to consistent explanations, focusing on clinically relevant variability^23^. Different methods may provide varied explanations due to data-related and model-related factors^8^. None-the-less, different explanations may point in the same direction.

When assessing consistency consider: 1) *sensitivity to design/parameters variations*, 2) *feature agreement at the cohort level*, and 3) *direction agreement at the patient level*^9^. Agreement can be measured using feature agreement^31^ and Spearman’s rank correlation coefficient like in ref. ^9^ or metrics like Cohen’s kappa and Krippendorf’s alpha^32^. Generating an explanation ensemble and quantifying confidence in XAIs can help reduce discrepancies.

**Note**: Vital signs inputs are often interrelated, meaning different algorithms might emphasize different features but point in the same direction or reflect the same physiology issue (e.g., respiratory rate and oxygen flow point out respiratory physiology issues).

***Item 8****. AI robustness:* Ensemble models combine predictions from multiple models to reduce variance and improve generalisation^33^. This can also apply to explanations by aggregating local explanations from different explainers using majority voting or weighted sums. Thus, it can help mitigate XAIs discrepancy and improve robustness.

**Note**: In time-sensitive applications, ensemble models may be impractical.

***Item 9****. Causal validity:* Causes as the main constituents of an explanation^34^. While popular XAI methods (e.g., SHAP^35^, LIME^36^, etc.) show correlations between features and outcomes, they don’t establish causal relationships. In ICU settings, where inputs like vital signs are highly interrelated, such explanations cannot reliably guide decisions. If used, explanations should be evaluated for alignment with causal baselines using quantifiable metrics such as absolute error, reciprocal rank, Spearman’s Rho, or Kendall’s Tau^37^. The level of explanation should match the level of medical knowledge drawn on during development. Where knowledge is limited in application, for example the mechanism is unknown, strong empirical evidence is needed to rely on causal models.

**Note:** For time-sensitive decisions, faster, non-causal methods (e.g., feature importance, saliency maps) may help, but should be used cautiously, and their limitations reported.

***Item 10****. Narrative reasoning:* Clinicians value explanations that narrate the patient’s clinical trajectory^14^. Main patient-level (local) explanations could help explore patient trajectories. For example, 30% of patients with low blood pressure triggering an early warning system had or developed sepsis, offering insights into potential underlying or consequent conditions. In not time-critical settings, counterfactual explanations could help identify the key factors influencing risk and suggest what changes could modify the outcome.

**Note:** While causal methods are most appropriate for investigating patients’ trajectories, popular XAI methods reveal correlations which may to some extent identify informative relationships between explanations and those trajectories.

## Model attributes

***Item 11****. Bias and fairness:* AI systems can behave unfairly for a variety of reasons^38^, including data under representation. Explanations can help identify model biases and improve fairness^25^. Global explanations can be used to identify systematic bias. If features such as LoS, age, race, gender, etc. have disproportionately high importance in determining outcomes, this could indicate the model is biased. Evaluating cases (true positive (TP), false positive (FP), true negative (TN), false negative (FN)) could help identify sources of bias or model errors^9,39^. Combining global explanations with fairness metrics like disparate impact or equalized odds can assess how fairly the model treats different groups. Tools like Fairlearn^40^ and TensorFlow’s Fairness^41^ can help explore fairness and bias.

***Item 12****. Model troubleshooting:* Users would want explanations for scrutability and debugging^18^. Analysing the data distributions of main contributors can help pinpoint the causes of common model failures^9^. However, the main contributors to correct (TP, TN) and incorrect (FP, FN) predictions may overlap. To analyse this, compute the frequency, means, and standard deviations of the top contributors for each case (TP, TN, FN, FP) and assess how incorrect predictions differ from correct ones. Note that while means may differ, the variability in subgroups can lead to overlap and incorrect predictions. For image data, visual inspection and comparison between the case is recommended at minimum. Person correlation or distance correlation could be used to quantify the similarity between heatmap vectors.

**Note:** Bootstrapping and comparing distributions help quantify feature importance uncertainty and assess the discriminative power of main contributors.

***Item 13****. Interpretation:* Summarise the key findings in the context of XAI and model audit, alongside previous studies. Appropriate reporting contributes trust calibration^23^. For example, explanations show low agreement with the ground truth (40%), but the primary contributors are considered clinically relevant by a domain expert.

***Item 14****. XAI limitations:* Report XAI performance and limitations clearly. Being aware of limitations helps calibrate the trust. Non-causal and XAI without strong empirical evidence cannot guide intervention choices and this should be stated explicitly. For example, explanations alone are insufficient to guide decisions. XAI is an adjunct that helps rationalise model outputs in a clinical context.

## Roadmap for scale-up and refinement

To support further improvement and generalisability and broader implementation of the checklist, we plan a structured roadmap involving broader expert validation, formal dissemination and field testing. The checklist will be registered with the EQUATOR Network to enable early adoption, early engagement from the community and address an immediate need for practical guidance on evaluating XAI in clinical contexts. A Delphi process is planned to encompass a broader range of internationally recognised institutions including universities, health tech companies, regulatory agencies, and clinical experts. This will establish wider consensus and enhance the checklist’s applicability across diverse clinical domains and use cases. Many of these stakeholders have already contributed to the development of XAI or represent key sectors involved in its implementation and oversight. This document will serve as formal base for further refinement and standardisation. Validation through multi-institutional trials represents a final step that is expected to emerge organically as the checklist is scaled and widely standardised.

## Conclusion

With the exponential growth in published papers intended for healthcare applications utilising XAI, adequate evaluation and transparent reporting are critical to gain empirical evidence on XAI capabilities with the aim of mitigating potential ethical and safety risks. To date no standardised XAI evaluation recommendations are available. Proposed XAI guidelines, incorporating the items and recommendations summarised in this CLIX-M checklist, will assist in achieving this aim and be a starting point for standardised evaluation of XAI. This Checklist, and the proposed guidelines, are primarily intended for researchers, AI-practitioners in software companies, journal editors and per reviewers. However, academic institutions, policy makers, regulators will also have direct benefits of increased quality in evaluation and reporting of XAI systems. It is important to acknowledge potential tension between some items. For instance, explanations may appear actionable without being causally valid, or domain-relevant features may introduce fairness concerns. Similarly, providing uncertainty details may cause cognitive overload under time pressure. If the only aim is model troubleshooting, clinical aspects may not be important. These trade-offs highlight the importance of contextual, purpose-driven evaluation and multi-stakeholder input. The CLIX-M checklist is a provisional tool that will now undergo further standardisation. In the meantime, it can be used to guide practicians in the evaluation and transparent reporting of deployed XAI methods to determine the true clinical potential of applied XAI.

## Supplementary information

Supplement_SubmissionRev1_Final

## Data Availability

No datasets were generated or analysed during the current study.
