# Supplementary material for: Clinician-informed XAI evaluation checklist with metrics (CLIX-M) for AI-powered clinical decision support systems
Source: NPJ Digit Med. 2025 Jun 14;8:364. doi: 10.1038/s41746-025-01764-2 (PMC12167368; doi:10.1038/s41746-025-01764-2)
Supplement: Supplementary file 1 — Supplement_SubmissionRev1_Final [file 41746_2025_1764_MOESM1_ESM.docx]

Clinician-Informed XAI Evaluation Checklist with Metrics (CLIX-M) for AI-Powered Clinical Decision Support Systems

| Supplementary Table1 CLIX-M checklist for the evaluation and reporting of studies including eXplainable AI (XAI). When using XAI in practice: a. Use clinical judgement to evaluate explanation coherence. b. Be aware of XAI limitations. c. Be aware of data quality used by XAI system. d. Treat XAI output only as an adjunct. | | | | | |
| --- | --- | --- | --- | --- | --- |
| **Item** | **Checklist item** | | **Item details** | **Phase*** | **Section†** |
| 1 | Purpose | Provide a summary of objectives, i.e. for which purpose explanations are developed and intended. | | D;E | M |
| **Clinical attributes** | | | |  |  |
| 2 | Domain relevance | Assess (see Table 2), report and discuss explanation actionability performed by the end users or the relevant domain literature. | | D;E | M;R;D |
| 3 | Coherence | Assess (see Table 2), report and discuss how the explanations agree with human rationales and how reasonable they are. | | D;E | M;R;D |
| 4 | Actionability | Assess (see Table 2), report and discuss explanation informativeness and potential to impact the workflow. | | D;E | M;R;D |
| **Decision attributes** | | | | | |
| 5 | Correctness | Assess, report and discuss the correctness of explanations benchmarked against the ground truth as it is done for quantifying predictive accuracy of a model. | | D;E | M;R;D |
| 6 | Confidence | Quantify, report and discuss confidence of deployed XAIs, i.e. explanations’ confidence scores. | | D;E | M;R;D |
| 7 | Consistency | Quantify, report and discuss XAIs 1) sensitivity on underlying design variations, 2) sensitivity on the deployed model by quantifying feature agreement at cohort level and 3) agreement on contribution direction by quantifying sign agreement at the patient level. | | D;E | M;R;D |
| 8 | XAI robustness | If ensemble XAI is used to improve explanation robustness provide details which methods are used and how final explanations are obtained. | | D;E | M;R;D |
| 9 | Causal validity | Discuss capability of deployed XAI to capture causal relationships. If it capable to capture casual relationships provide relevant details and how it is validated. | | D;E | D |
| **Machine attributes** | | | | |  |
| 10 | Narrative reasoning | Explore and report informative relationships between explanations and the patients’ trajectories (if appliable). | | D;E | R;D |
| 11 | Bias and Fairness | Report how explanations are used to explore model fairness and potential biases. Explain the findings, and whether it is used for further model improvement. | | D;E | R;D |
| 12 | Model troubleshooting | Explore data distribution of the main contributors for correct and incorrect predictions/decisions (TP, TN, FP, FN)**‡**. Report overlaps between correct and incorrect outputs and whether and how this information has been used for model improvement. | | D;E | R;D |
| 13 | Interpretation | Provide an overall interpretation of the main findings in the context of XAI and model audits, and related prior research. | | D;E | D |
| 14 | XAI limitations | Discuss XAI limitations. | | D;E | D |
| ***** D= items relevant only to XAI development; E=items relevant to the evaluation of a XAI in clinical settings  **†** M= items to be reported in the Methods section; R= items to be reported in the Result section; D= items to be reported in the Discussion section.  **‡** True positive (TP), true negative (TN), false positive (FP), false negative (FN) | | | | |  |

| Supplementary Table 2 \| Scales for clinical attributes: domain relevance, reasonableness, actionability. | | |
| --- | --- | --- |
| **Domain relevance** | **Coherent** | **Actionability** |
| Very irrelevant | Very incoherent | Not actionable |
| Irrelevant | incoherent | Slightly actionable* |
| Relevant | Coherent | Actionable |
| Very relevant | Very coherent | Highly actionable |
| * Vague or limited usefulness to action; might support decision-making but not reliably. For example, not all highlighted image areas are clinically meaningful or impact the workflow. However, some useful information can still be extracted and acted upon. | | |

CLIX-M Checklist: Guided Examples

**Hypothetical Example 1: Early Warning System (EWS) activation**

CDSS is designed to predict activation of an Early Warning Score (EWS) system, intended to guide pre-emptive clinical treatment planning ^1^. Considered input data: Vital signs, demographics data and patient consciousness. Where possible, results are drawn from actual (referenced) case studies. Predictive model: XGB. Considered explainer: SHAP^[[1]](#footnote-1)^. Considered phase: Development (D).

| Supplementary Table 3 Hypothetical Example 1: Early Warning System (EWS) activation | | | | | |
| --- | --- | --- | --- | --- | --- |
| **No** | **Checklist item** | | **Item details** | **Phase*** | **Section†** |
| 1 | Purpose | The explanations are intended to help clinicians understand the key contributors to the models decisions and to help the developer to audit the model’s behaviour ^1^. | | D | M |
| **Clinical attributes** | | | |  |  |
| 2 | Domain relevance | M: To investigate domain relevance of generated explanations, a clinician who is project collaborator was asked to evaluate the top 10 explanations at the cohort level using Likert scale.  R: “Very relevant”, was obtained for all considered variables. | | D | M;R;D |
| 3 | Coherence | M: Top 3 patient-level (local) explanations were assessed for coherence using a Likert scale across 10 randomly selected cases.  R: When a combination of vital signs was presented, the rater agreed on the score of “Very coherent” (Median=4). In one of the cases a patient who underwent surgery was identified at risk and would be likely to trigger EWS. Low respiratory rate was the main contributor along with oxygen saturation, supplemental oxygen delivery and low systolic blood pressure. This would inform the attending clinician that the mode of risk was respiratory failure, characterised by low respiratory rate, such as would occur with opiate excess.  In another example, particularly low systolic pressure with low temperature, low heart rate and oxygenation were recognised as a phenotype indicative of sepsis or infection.  Although Length of Stay (LoS) was among the top contributing factors in 30% of correctly predicted EWS activations, it was rated as “Incoherent”.  D: LoS may be valuable for modelling but not valuable as an explanation provided in the dashboard or explanation provided to clinicians to justify the model’s decision for activating the EWS ^5^. | | D | M;R;D |
| 4 | Actionability | M: Top 3 patient-level (local) explanations were assessed for actionability using a Likert scale across 10 randomly selected cases.  R: The rater strongly agreed the top features contributing to the prediction were at the border of “Actionable” and “Very actionable” (Median=3.5). Explanations based on unmodifiable or late-appearing indicators (e.g. LoS) were considered less useful for decision-making (Median = 1, “Not actionable”).  D: While LoS may be valuable for modelling it is not valuable as an explanation provided in the dashboard ^5^. | | D | M;R;D |
| **Decision attributes** | | | | | |
| 5 | Correctness | M: Correctness is calculated as the fraction for which explanations of the most contributing predictors match recorded red flag events. For instance, an explanation is scored as 1 if it correctly identifies the trigger and 0 if it does not. Aggregating these scores across all predictions allows for the calculation of explanation correctness, analogous to how predictive accuracy is measured ^4^.  R: Correctness was 0.80 (2). | | D | M;R;D |
| 6 | Confidence | M: To evaluate XAI module confidence, confidence intervals (CIs) were obtained with 100 bootstrapped samples drawn with replacement from the test dataset to compute global explanations for each resampled dataset.  R: Mean and CIs of the aggregated feature importance value for each feature is provided in the table below.   \| Variable \| Mean \| 95% CI \| \| --- \| --- \| --- \| \| SpO2 \| 1.2 \| [1, 1.4] \| \| Systolic Blood presssure \| 2.6 \| [2.2, 2.9] \| \| Heart rate \| 3.8 \| [3.5, 4.2] \| \| … \| … \| … \| | | D | M;R;D |
| 7 | Consistency | M: Consistency results are represented by an average agreement across 5 independent runs.  Top 5 contributors were used to test:  1) Sensitivity to design/parameters variations measured using feature agreement and Spearman’s rank correlation coefficient  2) Direction agreement at the patient level was measured using accuracy.  R: The results show high consistency for both tests when the top 5 contributors were considered ^4^. | | D | M;R;D |
| 8 | XAI robustness | M: Explanation ensembles were not explored in this study. | | **D** | M;R;D |
| 9 | Causal validity | SHAP values reflect association not causality. | | D | D |
| **Machine attributes** | | | | |  |
| 10 | Narrative reasoning | R: 40% of patients who triggered EWS due to elevated or decreased respiratory rate or low SpO2 had respiratory failure. | | D | R;D |
| 11 | Bias and Fairness | M: Global explanations were used to assess whether variables such as age, sex, or race had disproportionate influence on predictions.  R: Evaluation indicated no evidence of bias related to age, sex, or race. | | D | M;R;D |
| 12 | Model troubleshooting | R: Features identified as main contributors in correctly classified samples were also main contributors for the incorrect ones. Though means differ, the variability in the two subgroups is such that their ranges are not distinctly separate (2). | | D | R;D |
| 13 | Interpretation | Admin features, while relevant in broader context are less important in the context of actionability. Thus, though useful in development and modelling phase, they are not relevant for UX/UI design in this case. Explanations based on vitals may be useful in cases when they know little about a patient or to identify “at risk” patients who look deceptively stable (2). While counterfactual explanations could be beneficial, they are not suitable for the intended application. | | D | D |
| 14 | XAI limitations | SHAP values reflect correlations and may not indicate true causal drivers of deterioration. | | D | D |
| ***** D= items relevant only to XAI development; E=items relevant to the evaluation of a XAI in clinical settings  **†** M= items to be reported in the Methods section; R= items to be reported in the Result section; D= items to be reported in the Discussion section (if meaningful).  **‡** True positive (TP), true negative (TN), false positive (FP), false negative (FN) | | | | |  |

**Hypothetical Example 2: Radiology**

AI-enabled edema diagnoses using X-Ray ^6^. Considered input data: X-Ray Images. Where possible, results are drawn from actual (referenced) case studies. Model: Convolutional Neural Networks (CNN) Dense 121. Considered explainer: Grad-CAM. Considered phase: Evaluation (E).

| Supplementary Table 4 Hypothetical Example 2: Radiology | | | |  |  |
| --- | --- | --- | --- | --- | --- |
| **No** | **Checklist item** | | **Item details** | **Phase*** | **Section†** |
| 1 | Purpose | XAI was deployed to explore capabilities to rationalise the decision of AI model output and boost clinician’s trust. | | E | M |
| **Clinical attributes** | | | |  |  |
| 2 | Domain relevance | M: To investigate domain relevance of generated explanations, saliency maps were shown to 3 radiologists to evaluate domain relevance cohort level using Likert scale. Descriptive statistics using median for measuring central tendency was used.  R: Although the XAI highlighted non-relevant regions alongside relevant ones at the cohort level, it was still considered relevant to the clinical domain, with a median rating of 3 ('Relevant').  D: Explanations although pointing to irrelevant parts were still overall rated relevant. | | E | M;R;D |
| 3 | Coherence | M: Explanations were assessed for coherence using a Likert scale across 10 randomly selected cases. Grad Saliency method was used to generate heat maps. Threshold to each heat map was determined using Otsu’s method which iteratively searches for a threshold value that maximizes interclass pixel intensity variance to produce binary segmentations ^6^.  R: Median was 2.5, which lies between ‘Incoherent’ and ‘Coherent’, suggesting mixed perceptions of coherence. | | E | M;R;D |
| 4 | Actionability | R: In terms of actionability, shown explanations were assessed as “Slightly Actionable” (Median = 2).  D: Though overall explanations were vague, some useful information still could be extracted and acted upon. | | E | M;R;D |
| **Decision attributes** | | | | | |
| 5 | Correctness | M: Two evaluation metrics were used to compare generated segmentations to benchmarks ^6^. mIoU, measures how much, on average, either the saliency method or benchmark segmentations overlapped with the ground-truth segmentations. Hit rate, is a less strict metric that does not require the saliency method or benchmark annotators to locate the full extent of a pathology. A ‘hit’ indicates that the correct region was located regardless of the exact bounds of the binary segmentations. Localisation performance is then calculated as the hit rate across the dataset. We report the means of these metrics (mIoU and hit rate) over 1,000 bootstrap replicates on the test set, along with the 95% CIs using the 2.5th and 97.5th percentiles of the empirical distribution. ^6^  R: Mean localisation performance for edema pathology on test data was 0.362 for mIoU and 0.746 hit rate ^6^. | | E | M;R;D |
| 6 | Confidence | R: CIs for mIoU and hit rate are (0.35, 0.39) and (0.64, 0.84), respectively ^6^. | | E | M;R;D |
| 7 | Consistency | M: To test sensitivity to design/parameters variations, the model was run five times to generate explanations five times. Pearson correlation between flattened heatmap vectors was computed to quantify the variability. Variability is represented with mean and standard deviation (STD).  R: The results show moderate consistency with high variability (mean 0.49, STD 0.3). | | E | M;R;D |
| 8 | XAI robustness | M: We use an ensemble of checkpoints to create saliency maps to maximize model performance ^6^. We computed the saliency maps using the relevant checkpoints. We then take the mean of the saliency maps to create the final set of maps for the ensemble model.  R: Ensembles performed better than any individual method ^6^. | | E | M;R;D |
| 9 | Causal validity | Grad-CAM values reflect association not causality and cannot be used to guide intervention. | | E | D |
| **Machine attributes** | | | | |  |
| 10 | Narrative reasoning | NA | | E | R;D |
| 11 | Bias and Fairness | M: To test for bias, explanations at the cohort level were generated for different patient demographics, i.e. males and females, different ethnic groups.  R: Relevant regions did not significantly vary across different patient demographics, suggesting minimal bias. | | E | M;R;D |
| 12 | Model troubleshooting | M: To perform troubleshooting, explanation maps were generated for true positive (TP), true negative (TN), false positive (FP), false negative (FN) cases and visually compared.  R: Common partial overlap was present among all cases indicating no significant relevance for any of the cases. | | E | M;R;D |
| 13 | Interpretation | Change in model parameters lead to large variations in the heatmap suggesting high sensitivity and instability. Mixed perception suggests poor clinical validity and usability. There was statistically insignificant difference in localisation performance between the expert and the XAI (2). However, explanations while boosting trust in the AI also introduced automation bias ^7^. | | E | D |
| 14 | XAI limitations | Grad-CAM highlights areas correlated with the model’s prediction, not necessarily the causal factors. This means that highlighted areas do not indicate true clinical importance or validity. While Grad-CAM performed well for larger and simpler targets, its localisation accuracy declined significantly for smaller or more complex-shaped pathologies, showing the greatest gap compared to human performance in these cases. (2). | | E | D |
| ***** D= items relevant only to XAI development; E=items relevant to the evaluation of a XAI in clinical settings  **†** M= items to be reported in the Methods section; R= items to be reported in the Result section; D= items to be reported in the Discussion section (if meaningful).  **‡** True positive (TP), true negative (TN), false positive (FP), false negative (FN) | | | | |  |

References

1. Brankovic, A. *et al.* Explainable machine learning for real-time deterioration alert prediction to guide pre-emptive treatment. *Sci. Rep.* **12**, 11734 (2022).

2. Di Martino, F. & Delmastro, F. Explainable AI for clinical and remote health applications: a survey on tabular and time series data. *Artif. Intell. Rev.* **56**, 5261–5315 (2023).

3. Caterson, J., Lewin, A. & Williamson, E. The application of explainable artificial intelligence (XAI) in electronic health record research: A scoping review. *Digit. Health* **10**, 20552076241272657 (2024).

4. Brankovic, A., Cook, D., Rahman, J., Khanna, S. & Huang, W. Benchmarking the most popular XAI used for explaining clinical predictive models: Untrustworthy but could be useful. *Health Informatics J.* **30**, 14604582241304730 (2024).

5. *Brankovic A, Rahman J, Delaforce A, Bradford D, Li J, Magrabi F, Cook D (2024) Mitigating Ethical Risks in the Development of Artificial Intelligence (AI)-Enabled Tools with Explainable AI (XAI) Component. CSIRO, Australia.*

6. Saporta, A. *et al.* Benchmarking saliency methods for chest X-ray interpretation. *Nat. Mach. Intell.* **4**, 867–878 (2022).

7. Cabitza, F. *et al.* Rams, hounds and white boxes: Investigating human–AI collaboration protocols in medical diagnosis. *Artif. Intell. Med.* **138**, 102506 (2023).

1. SHAP was selected as the most frequently used explainability method ^2,3^ although the study ^4^ report results also for DTD. [↑](#footnote-ref-1)
